# Study protocol of the ‘HEAL-HOA’ dual randomized controlled trial: Testing the effects of volunteering on loneliness, social, and mental health in older adults^☆^

**DOI:** 10.1016/j.conctc.2024.101275

**Published:** 2024-02-15

**Authors:** Lisa M. Warner, Da Jiang, Dannii Yuen-lan Yeung, Namkee G. Choi, Rainbow Tin Hung Ho, Jojo Yan Yan Kwok, Youqiang Song, Kee-Lee Chou

**Affiliations:** a Department of Psychology, MSB Medical School Berlin, Rüdesheimer Straße 50, 14197, Berlin, Germany; b The Education University of Hong Kong, 10 Lo Ping Rd, Tai Po, Hong Kong; c Department of Social and Behavioural Sciences, City University of Hong Kong, Tat Chee Ave, Kowloon Tong, Hong Kong; d Steve Hicks School of Social Work, University of Texas at Austin, 1925 San Jacinto Blvd, Austin, TX, USA; e Department of Social Work & Social Administration, Centre on Behavioral Health, The University of Hong Kong, Pokfulam, Hong Kong; f School of Nursing, The University of Hong Kong, Pokfulam, Hong Kong; g Department of Biochemistry, The University of Hong Kong, Pokfulam, Hong Kong

**Keywords:** Loneliness, Older adults, Volunteering, Perceived social support, RCT, Civic engagement, Social network, Anxiety, Depression, Stress, Sleep, Cortisol, Intervention

## Abstract

**Background:**

Interventions to reduce loneliness in older adults usually do not show sustained effects. One potential way to combat loneliness is to offer meaningful social activities. Volunteering has been suggested as one such activity – however, its effects on loneliness remain to be tested in randomized controlled trials (RCT).

**Methods:**

This planned Dual-RCT aims to recruit older adults experiencing loneliness, with subsequent randomization to either a volunteering condition (6 weeks of training before delivering one of three tele-based loneliness interventions to older intervention recipients twice a week for 6 months) or to an active control condition (psycho-education with social gatherings for six months). Power analyses require the recruitment of N = 256 older adults to detect differences between the volunteering and the active control condition (128 in each) on the primary outcome of loneliness (UCLA Loneliness Scale). Secondary outcomes comprise social network engagement, perceived social support, anxiety and depressive symptoms, self-rated health, cognitive health, perceived stress, sleep quality, and diurnal cortisol (1/3 of the sample). The main analyses will comprise condition (volunteering vs. no-volunteering) × time (baseline, 6-, 12-, 18-, 24-months follow-ups) interactions to test the effects of volunteering on loneliness and secondary outcomes. Effects are expected to be mediated via frequency, time and involvement in volunteering.

**Discussion:**

If our trial can show that volunteers delivering one of the three telephone-based interventions to lonely intervention recipients benefit from volunteer work themselves, this might encourage more older adults to volunteer, helping to solve some of the societal issues involved with rapid demographic changes.

## 1 Introduction

Loneliness has been described as social distress, arising when a discrepancy between one's desired and perceived social relationships is regarded as encompassing *behavioral* components such as withdrawal, as well as *motivational* forces to reconnect [1]. Loneliness is highly prevalent across different life phases [2], but might have a stronger impact on the mental and physical health of older, rather than younger, adults [3,4]. Older adults are less likely to recover from feeling lonely [5]. In a longitudinal survey in Hong Kong in 2018, 36% of older adults reported feeling lonely *sometimes* and 10.1% answered *always* feeling lonely [6]. The COVID-19 pandemic and its associated public health measures increased the risk of older adults experiencing loneliness against the background of additional risk factors such as possessing a low-income, living alone, and having limited access to the internet [[7], [8], [9]]. Therefore, interventions aiming to reduce loneliness in older adults, as well as structures implementing these interventions, must not only be effective, but also scalable and implementable in practice (preferably without physical contact). One potentially scalable opportunity to combat loneliness among older adults is to offer meaningful social activities, such as volunteering [10].

### 1.1 Benefits of volunteering for older adults

Volunteering has been defined as “… any activity in which time is freely given to benefit another person, group or cause.” [11]. According to the ‘social model for health promotion for an aging population’ [12], engaging as a volunteer may increase social, cognitive, and physical activities, which positively affect functional (e.g., balance), physiological (e.g., cortisol), psychosocial (e.g. social support), and cognitive mechanisms. This ultimately leads to better physical functioning and health. Although these theoretically assumed benefits of volunteering have been well-documented in longitudinal studies, some long-term surveys found no effect of volunteering on older adults' well-being. Some findings also suggested reverse causality [e.g., higher rates of volunteering among those with higher levels of well-being, [13,14]], with only a few randomized controlled trials having been conducted to begin accumulating causal evidence [for reviews see Refs. [[15], [16], [17], [18]]]. For example, two US-based “Experience Corps” Trials [12] tested the effects of volunteering in schools against wait-list control groups, in samples of older adults. Among other outcomes, the program improved depressive symptoms, social network indices, functional impairment, self-reported physical strength, objectively measured walking speed, executive functioning, verbal learning, and memory [19,20]. In a previous trial among older adults in Hong Kong, we provided initial evidence that a psychological intervention aiming to increase motivational and volitional factors to start volunteering not only increased the duration of self-organized volunteering but also indirectly affected psychological measures [e.g., depressive symptoms; see Refs. [21,22]. Apart from these Experience Corps trials and our trial, only a handful of RCTs have tested the benefits of volunteering on health and well-being in late life [23,24]. Therefore, current evidence is small, often suffers from methodological limitations (e.g., no randomization, selection problems), and mainly stems from the U.S.A. [[16], [17], [18],^25^].

### 1.2 Effects of volunteering on loneliness, social, and mental health

**Fig. 1:**
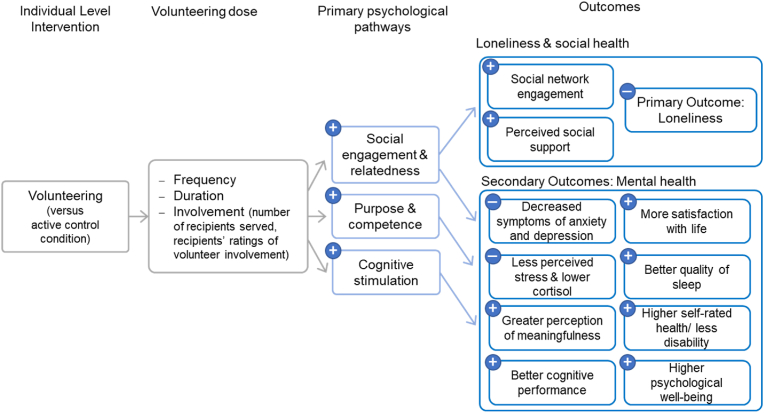
“Conceptual framework for effects of volunteering on loneliness social and mental health” in older adults tested in the HEAL-HOA trial (https://osf.io/2k5ep/).

Numerous theories proposing determinants of loneliness exist, with only few naming potentially effective interventions for loneliness reduction [1]. Our trial is based on Hawkley's model on the ‘effects of loneliness on health and loneliness reduction approaches’, which proposes that individual and community level interventions, such as volunteering may reduce loneliness and associated health deterioration [10]. Hawkley proposes that loneliness directly deteriorates physical, emotional, and mental health as well as decreases health behaviours (e.g., sleep hygiene), which can result in increased mortality. On the other hand, frameworks such as the ‘social model for health promotion for an aging population’ [12] or the ‘theory of change for volunteer well-being model’ [26], propose that volunteering supports the activation of several psychological pathways to improved mental and physical health. Four out of the six observational studies in a review found levels of loneliness to be lower in older adults who volunteer [24]. Because both lines of theoretical reasoning and empirical research suggest that volunteering has a positive effect on loneliness in older adults, this planned dual-RCT aims to test the effects of volunteering primarily on loneliness, but also on further social and mental health indices in older adults in Hong Kong (see conceptual framework in Fig. 1). To date, no randomized controlled trials have been conducted to test the effects of volunteering on loneliness [23]. In addition to assessing the effects on volunteers' health and well-being, the feasibility and scalability of our dual approach of training older adults as lay counselling volunteers to deliver loneliness interventions to even older, lonely, poor and isolated recipients is of public health interest.

### 1.3 Using lay volunteers to deliver loneliness interventions via telephone

Given the global shortages of professional mental health workers, non-professional delivery of mental health interventions is an important area of research. Unlike other psychotherapeutic approaches that rely heavily on mental health clinicians, studies have demonstrated that simple and structured programs can be delivered by trained volunteers or lay counselors with a bachelor's degree [[27], [28], [29], [30]]. Therefore, our aim is also to evaluate whether lay retirees can be trained to successfully deliver telephone interventions, aiming to reduce loneliness in older adults in Hong Kong, and whether this method is scalable for future situations that may require physical distancing [the RCT to assess the effects on intervention recipients can be found in Ref. [31]].

The in-person delivery of interventions to reduce loneliness in older adults poses some significant challenges: Many older adults face mobility and transportation problems. Deploying interventionists for in-home sessions with older adults is costly, and therefore not sustainable and scalable. In light of these challenges of in-person delivery, recent intervention programs have successfully used video-conferencing, telephone, or smartphone apps as a way to reduce loneliness and social isolation in older adults [28,[32], [33], [34], [35], [36], [37]].

### 1.4 Study objectives and hypothesis

**Fig. 2:**
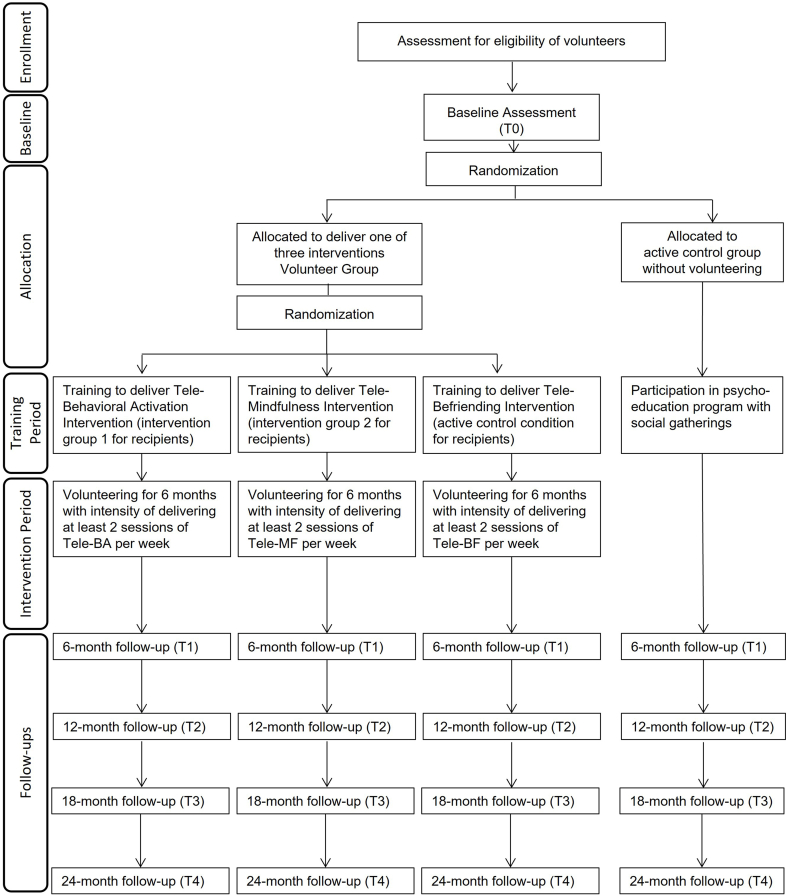
CONSORT Flow-Chart depicting two allocations and four conditions.

The proposed study is a dual-RCT aiming to: a) evaluate the effects of volunteering on older adults, delivering psycho-social interventions (volunteer trial); and b) test the effects of these interventions on older intervention recipients’ experienced loneliness [recipient trial see separate study protocol see Ref. [31]]. We aim to provide evidence that engagement in volunteering conveys benefits for older *volunteers* delivering interventions to reduce loneliness. This RCT randomizes retirees into the volunteering condition, in which one of the following tele-interventions are delivered: (1) behavioral activation; (2) mindfulness; and (3) befriending (active control for intervention recipients). These volunteer conditions will be compared against an active control condition (psycho-education related to enhancing mental, physical and cognitive health after retirement) with social gatherings. See Fig. 1 for a conceptual framework of expected mechanisms and effects of volunteering in our trial, Fig. 2 for an overview of randomized conditions, and study design and Table 1 for exact measures and time-points.

**Table 1:** SPIRIT schedule of enrolment, conditions, and assessments.

	STUDY PERIOD							
	Enrolment	Allocations	Post-allocation					
					Follow-ups at months			
TIMEPOINT	*−1*	*Baseline (T0)*	*6 weeks volunteer training vs. psycho-education*	6-months *volunteering vs. no-volunteering*	*6 (T1)*	*12 (T2)*	*18 (T3)*	*24 (T4)*
ENROLMENT:								
Eligibility screen (UCLA Revised Loneliness Scale)	✓							
Informed consent	✓							
Allocations at random (1. volunteering vs. no-volunteering) (2. within volunteering condition delivery of one out of three tele-based interventions to recipients 1) mindfulness, 2) behavioral activation, 3) befriending)		✓ ✓						
CONDITIONS:								
*Volunteering*								
*Active Control*								
ASSESSMENTS:								
*Socio-demographics/Covariates*								
age; gender; number of children; living arrangements (number/role of co-habitants); education level		✓						
marital status, chronic diseases [38]		✓				✓	✓	✓
employment status; personal income		✓			✓	✓	✓	✓
*Mediators*								
Frequency and duration of volunteering, forms filled in by volunteers on number of sessions delivered and recipients served		✓			✓	✓	✓	✓
Rating by recipients on “effectiveness as interventionist” for each volunteer treatment evaluation inventory [39]				✓				
*Primary Outcome*								
UCLA Revised Loneliness Scale [40,41]		✓			✓	✓	✓	✓
*Secondary Outcomes*								
De Jong Gierveld Loneliness Scale [42,43]		✓			✓	✓	✓	✓
Multidimensional Scale of Perceived Social Support [44,45]		✓			✓	✓	✓	✓
Lubben Social Network Scale [46,47]		✓			✓	✓	✓	✓
Patient Health Questionnaire-9 [48,49]		✓			✓	✓	✓	✓
Hospital Anxiety and Depression Scale [50,51]		✓			✓	✓	✓	✓
Perceived Stress Scale [52,53]		✓			✓	✓	✓	✓
Meaningful/happy events [31]		✓			✓	✓	✓	✓
Satisfaction with Life Scale [54,55]		✓			✓	✓	✓	✓
Psychological Well-Being Scale [56,57]		✓			✓	✓	✓	✓
Montreal Cognitive Assessment [58,59]		✓				✓	✓	✓
MoCA 5-min protocol [58,59]					✓			
General self-rated health, vision, hearing [31]		✓			✓	✓	✓	✓
WHO Disability Assessment Schedule 2.0 [60,61]		✓			✓	✓	✓	✓
Sleep Condition Indicator [62,63]		✓			✓	✓	✓	✓
Diurnal cortisol (see table notes)		✓			✓	✓	✓	✓

Table notes: Diurnal cortisol: After providing detailed guidelines in saliva sample collection, one third of the sample is invited to collect three saliva samples themselves using Salivette tubes at home on two consecutive weekdays, three times each day: after waking up, before lunch, and before bedtime. The collected samples are kept frozen, and the cortisol levels are determined according to the manufacturer's standard using an ELISA kit (Salimetrics, PA, USA).

The following hypotheses will be tested in the dual-RCT “Helping Alleviate Loneliness in Hong Kong Older Adults (HEAL-HOA)”:

1. Primary outcome: Compared to the active control condition (psychoeducation and social gatherings), volunteering to deliver interventions against loneliness (i.e., volunteering condition) would result in decreased levels of loneliness also for volunteers at 6, 12, 18 and 24 months.
2. Secondary outcomes: The volunteering condition is also expected to show decreased perceived stress, diurnal cortisol levels, sleep problems, symptoms of anxiety, and depression, as well as increased levels of social network engagement, perceived social support, satisfaction with life, self-rated health at 6-, 12-, 18-, and 24-month follow-up, compared to those assigned to the active control condition,
3. Mediation effects: To test whether the training to deliver tele-based counselling against loneliness is the active ingredient for more positive outcomes in the volunteer condition, or whether volunteering itself is the mechanism of action, we will also test potential mediation effects of amount and intensity (e.g., frequency, time, involvement rated by recipients, meaningfulness of events, number of recipients served) of volunteering.
4. Moderation effects: Recent observational studies suggested that volunteering might not only directly link to health outcomes, but act as a potential moderator on the association between stress and social/mental health [[64], [65], [66], [67]]. Therefore, these indirect effects will be tested exploratively.
5. We also aim to detect optimal levels of volunteering for different subgroups (e.g., volunteers with better physical and mental health).

## 2 Material and methods

### 2.1 Research Integrity

Ethical approval for this proposed project was obtained through the Human Research Ethics Committee of the Education University of Hong Kong (reference number: 2019-2020-0442). The study was prospectively registered in the Clinical Trials Registry of the University of Hong Kong Clinical Trials Centre (number HKUCTR-2929; as well as retrospectively registered at the Chinese Clinical Trial Registry in the WHO Registry Network, number ChiCTR2300072909; the content of both registrations is identical).

### 2.2 Volunteer recruitment and eligibility

This study targets community-dwelling Chinese adults living in Hong Kong. The inclusion criteria for volunteers are: 1) aged between 50 and 70 years; 2) consent to be trained and serve at least 2 h per week for 6 months; 3) possessing at least 3 years of education in secondary school; 4) reporting no major physical and mental health problems, or cognitive impairment; 5) no current full- or part-time employment; 6) engagement in formal volunteering ≤4 times within the last year; and 7) 3-item UCLA Loneliness Scale score of ≥6. Potential volunteers have been recruited through community centers for older adults, older adult academies, and from public housing estates. Currently, follow-up assessments are under way. The study's purpose, time commitment for participation, voluntariness, right to quit without negative consequences, and inclusion criteria are clearly presented before a volunteer gives verbal consent to participate. Potential volunteers who meet the above criteria are subsequently asked to sign a written informed consent form. Participants receive 200 HKD (approx. 25 USD) as compensation for their participation, as well as a certificate and an additional 50 HKD if they serve more than six intervention recipients.

### 2.3 Sample size

A required sample size for the RCT on loneliness was estimated for one of the primary outcomes, that is, loneliness assessed through the UCLA Loneliness Scale [68]. To date, there is cross-sectional and longitudinal evidence for a relation between volunteering and loneliness, but to our knowledge no experimental evidence exists [only one trial under way, see Ref. [23]]. Previous trials testing loneliness interventions in older adults yielded an effect size of 0.5 [32]. We estimate the sample size based on a smaller effect size, i.e., 0.2, as the focus will be placed on *volunteers* delivering the interventions, not the *participants* of loneliness interventions. It is also assumed that volunteers will enter the trial with elevated but not extreme levels of loneliness (according to the inclusion criteria). For the RCT on volunteering, power analyses revealed that a total sample size of 256 is required to detect differences among any of the three volunteering intervention conditions versus the active control condition (psycho-education with social gatherings), using a multivariate analysis of covariance with an effect size of 0.2, a power of 90%, and an alpha level of 0.05, with 128 individuals in each condition. Considering the drop-out rate of 15% over a period of 12 months, the target sample size is adjusted to 302 (with 151 individuals in each condition).

### 2.4 Study design and randomization

Eligible potential volunteers are randomized into the volunteering condition or into the active control condition, which consists of a psycho-education program with social gatherings to ensure the same contact to the research team as in the volunteer condition. Those randomized into the volunteering group are again randomized to serve for 6 months to deliver *one* of the three interventions. Online random numbers, generated by a research assistant, are used for both randomizations. Volunteers’ primary and secondary outcome measures are assessed at baseline, 6-, 12-, 18- and 24-months follow-up. An overview of the study design can be found in the CONSORT Fig. 2. Due to the nature of the intervention, volunteers cannot be blinded as the types of activities in each intervention group are distinct.

### 2.5 Volunteer training

An experienced social worker (to train Tele-BA), an experienced mindfulness practitioner (to train Tele-MF) and one research assistant (to train Tele-BF) deliver training sessions for volunteers, separately, according to their respective volunteer condition (see below, details and behavior change techniques used see OSF supplement: https://osf.io/fmuqe). Each volunteer is required to attend six weekly training sessions (2 h per week), before they independently deliver the tele-intervention to older intervention recipients. These training sessions, which include role-playing and other practical exercises, take place in a small face-to-face interactive group format, with four to six volunteers in each session. The research team works closely with the trainers to prepare PowerPoint presentations and video-tape selected sessions, in order to ensure comparability of the training content across small groups within the same intervention program. Volunteers do not know which interventions are hypothesized to be more effective for intervention recipients.

### 2.6 Volunteer and non-volunteer conditions

#### 2.6.1 Volunteer condition

Volunteers deliver one of three evidence-based loneliness interventions to intervention recipients via telephone: 1) Tele- Behavioral Activation [Tele-BA, adapted from Ref. [69]], 2) Tele-Mindfulness [Tele-MF, adapted from Ref. [70]], or 3) Tele-Befriending, as the active control condition [Tele-BF, adapted from the National Ageing Research Institute [71]]. Each intervention is delivered to intervention recipients in eight sessions, with two sessions per week of approximately 30 min each, resulting in a 4-week intervention period for each participant. Volunteers can deliver up to four intervention sessions per week via telephone, for up to two intervention recipients, and are scheduled to deliver one of the interventions over a period of 6 months. Total volunteering time will range between 60 and 120 min and two to four weekly contacts with intervention recipients during these 6 months. Details on the content of these intervention sessions can be found in the protocol for the intervention recipient-arm of this dual-RCT (see Ref. [31] and on OSF: https://osf.io/fmuqe). In summary, those in the volunteer intervention conditions are asked to participate in at least 60 min of formal volunteering (delivering one of three interventions via telephone) per week, for at least 6 months as part of this study. Individuals can continue to serve as volunteers beyond the 6 months, but may also choose to stop delivering the interventions, choose a different volunteering activity outside of the study or choose to stop volunteering altogether.

#### 2.6.2 Non-volunteer condition

The active control condition consists of a psycho-education program. Individuals in the active control condition receive six 2-h face-to-face sessions (or Zoom sessions, depending on the pandemic regulations) of a psycho-education program on the topics of retirement and healthy behaviors. The program aims to help individuals manage life after retirement and foster health behaviors. These meetings are comparable in frequency and in duration to the training sessions to become volunteer interventionists in the experimental groups of this RCT. Individuals in the active control condition are neither discouraged nor encouraged to take up any form of volunteering. Hence, individuals in this condition are free to volunteer outside of the study if they choose to do so. Volunteering frequency and duration will be assessed in this group as well. Therefore, mere-measurement effects (increased volunteering due to answering the questionnaire items on volunteering several times) can be controlled for.

### 2.7 Measures

Structured questionnaires in their validated Chinese versions are used to assess primary and secondary outcomes at baseline, 6, 12, 18 and 24 months after the volunteer period. Table 1 shows an overview of time-points and measures. Details can be found in the protocol by Jiang et al. [31].

#### 2.7.1 Mediators

**Number of intervention recipients served and sessions delivered**. In order to assess the extent to which the volunteers adhere to the protocols, as well as to monitor time and frequency of participation and volunteering within the trial condition, each volunteer is required to fill out a record form (i.e., date and duration of each call) after each contact with an intervention recipient. The research assistants use these data to calculate the number of sessions delivered and the number of intervention recipients served. The data are also used to assess adherence to the scheduled sessions, whether the protocol has been followed, and/or if any difficulties were encountered (the frequency of checking is often higher for each volunteer's first intervention case).

**Frequency and duration of volunteering.** Engagement in any volunteer activity (also outside the trial) is assessed by first giving a short definition of formal volunteering: “By <volunteering> we mean performing any form of unpaid and voluntary work in an organization (such as a nonprofit organization, sports club, or volunteer organization or group and providing services to others over the course of participating in this research). Informal help to neighbors, friends, and family (such as caring for one's grandchildren) is not counted as volunteer work in this questionnaire. However, if you formally take care of other people's children as part of a voluntary agency, this will be counted as voluntary work for the purpose of this questionnaire.” Three items based on Ayalon [72] are subsequently assessed, which translate to: 1) “In the past 4 weeks, have you ever volunteered?” Answers are dichotomous to filter for those who would answer the next two items and those who would skip them (0 = not at all, 1 = yes). 2) “Over the past 4 weeks, if you have participated in voluntary work on *a regular basis*, you spent _____ times every ____ days and spent ____ hours each time in voluntary work.” Frequency and hours are entered in the open text fields. 3) “Over the past 4 weeks, if you have participated in voluntary work *irregularly*, on average you spent _____ hours per week on voluntary work.” Based on these items, a frequency score and total volunteering minutes over the last 4 weeks can be derived.

**Volunteer involvement.** Intervention recipients of the tele-interventions will be asked to rate their satisfaction with their respective volunteer-interventionists and the intervention sessions at the first post-intervention measurement point. Performance of their respective volunteer will be rated with the two items “How engaged do you think the volunteer was during the intervention?” ranging from 1 (not engaged) to 7 (very engaged) and “How satisfied are you with the volunteer's training?” ranging from 1 (not satisfied) to 7 (very satisfied). In addition, recipients will fill in 11 items (e.g., “How effective do you think this intervention is?”) of the treatment evaluation inventory [TEI with different answering options from 1 to 7, with higher scores denoting a better evaluation [39]]. These scores can be used as an indication of involvement or effort volunteers put into their intervention delivery. Volunteers who delivered the intervention to more than one recipient will have multiple ratings, which will be averaged.

#### 2.7.2 Primary outcome

**Loneliness** is the primary outcome of the intervention and is measured by the 20-item Revised UCLA Loneliness Scale [40]. The Revised UCLA Loneliness Scale [41] comprises 20 items to assess loneliness and has been validated among Chinese older adults [40]. This version improved from the original UCLA Loneliness Scale [73] by addressing concerns regarding the direction of the items, discriminant validity, and social desirability [41]. Each item in the Revised UCLA Scale is a question starting with “How often do you feel …”. Participants indicate how often they think each question describes their experiences on a 4-point scale (1 = never to 4 = always). Sample items include “How often do you feel alone?” and “How often do you feel that there is no one you can turn to?”. Items are averaged, with higher scores indicating higher perceptions of loneliness.

To also differentiate between social and emotional loneliness, the De Jong Gierveld Loneliness Scale [42] will also be administered. The validated Chinese version of the 6-item De Jong Gierveld Loneliness Scale [42,43], is adopted to measure retrospective loneliness. Participants are asked to indicate their level of agreement with the six statements on a 3-point scale (1 = yes, 2 = more or less, 3 = no). Sample items are: “I experience a general sense of emptiness”, “Often, I feel rejected” or “I miss having people around.” The total score represents higher loneliness with higher values (after recoding reversely formulated items, ranging from 0 to 6).

#### 2.7.3 Secondary outcomes

The secondary outcomes include perceived social support, social network engagement, symptoms of anxiety and depression, satisfaction with life, psychological well-being, meaningful/happy events, cognitive health, self-rated health, functional health, perceived stress level, sleep quality, and diurnal cortisol. Measurement points and references can be found in Table 1, scoring details and sample items can be found in Jiang et al. [31].

### 2.8 Planned statistical analysis of the effects of the intervention

#### 2.8.1 Planned preliminary analyses

Descriptive statistics, correlation analysis, attrition analysis (t-tests to compare the socio-demographic and outcome variables of participants who drop out and those who complete the volunteer and active control period), and randomization tests (one-way analysis of variance [ANOVA]) will be conducted to test for differences in socio-demographic and outcome variables between conditions.

#### 2.8.2 Planned analyses of the effects of volunteering

The effects of volunteering on loneliness will be assessed using linear mixed models for repeated measures. Specifically, linear mixed models (LMMs, multilevel modeling with random intercept) that adjust for the dependency of repeated observations (baseline, 6, 12, 18, 24 months) will be used to determine interactions of within-subject timepoint x between-subject condition (volunteering versus non-volunteering), while controlling for potential confounders. For a sensitivity analysis, a similar repeated measures ANOVA model will be used. In case demographic characteristics show significant differences across groups at baseline, these will be controlled for in the models. An “intention-to-treat” analysis will be performed to assess differences in outcomes by incorporating participants who do not remain in the study until the final point of assessment. In addition to testing the effects of volunteering versus no volunteering (i.e., the active control condition of psycho-education and social gatherings), we will exploratively analyze the differential effects that might emerge in delivering one of three different loneliness interventions to intervention recipients. Intervention effects of volunteering on primary and secondary outcomes are expected to be mediated (indirect effects with bootstrapping) via frequency, duration (in or outside of the study), and involvement in volunteering (number of intervention recipients served, recipients’ ratings of volunteer involvement). Curvilinear effects will be explored on frequency, time, the number of intervention recipients to attend to at a time, and participant-rated involvement to detect possible trade-off effects of over-engagement. Given the moderation effects of volunteering on stress-health relations in recent studies, another objective of this study is to examine the moderating role of volunteering on the association between loneliness and perceived stress and cortisol, as well as on other secondary outcomes. Loneliness levels at the start of the study will operate as a further possible moderator for intervention success for both volunteers as well as intervention recipients of this dual-RCT.

## 3 Discussion

Although observational studies found associations between volunteering and lower mortality [74], research is far from establishing scientific evidence to prove a positive, causal effect of volunteering on health. However, it is commonly believed that volunteering not only contributes to society through supporting social services and charitable organizations, but also positively influences the mental and physical well-being of volunteers – especially later in life. Observational studies highlight improvements across various aspects associated with being a volunteer, e.g., increased social support [12], greater social contact [75], opportunities to expand one's social network [25], better mental and physical well-being [[15], [16], [17]]. However, whether volunteering can reduce experiences of loneliness remains to be tested.

The strengths of the HEAL-HOA trial are the randomization, large sample size, and standardization of intervention delivery, as well as the comparison of a volunteering condition versus an active control group. In addition to self-reports, we will also assess diurnal cortisol as an additional objective stress marker.

### 3.1 Risks and possible limitations of the planned study

One possible limitation of this dual-RCT is that both intervention recipients and those who deliver the loneliness interventions (volunteers) are selected according to elevated levels of loneliness. If loneliness is accompanied by behavioral and psychological (possibly self-sustaining) mechanisms as hypothesized by some authors (e.g., shyness, less socially competent behavior), matching lonely volunteers with lonely intervention recipients might not lead to optimal outcomes [1]. Therefore, the effects of the loneliness intervention and the delivery of the intervention will be tested at different levels of loneliness, post-hoc.

The contact between volunteers and intervention recipients will take place solely via telephone. This can be seen as a strength, as it enables volunteers to deliver tele-interventions to everyone in their time zone and language, while also being suitable for future crises, such as another pandemic. However, contact via telephone reduces social contact to verbal content only, without the possibility of physical touch or a way to interpret non-verbal cues. This bears the risk that the conversation is experienced as less deep and emotionally gratifying. The interventions are less likely to train the social skills of volunteers and intervention recipients who might particularly miss the “full-social-experience”, as these individuals all self-identify as relatively lonely at the start of the trial.

All three intervention programs delivered by volunteers educate them on how to cope with their own loneliness and stress. This potentially sensitizes volunteers to the benefits of activating their social networks and choosing to engage in social activities that positively affect their own social well-being. By teaching these strategies to intervention recipients, volunteers rehearse communicating socially rewarding strategies and are more likely to implement these strategies themselves. In order to test whether the training to deliver interventions is effective or rather the amount of volunteering and involvement in delivering the interventions, mediation analyses are planned.

The delivery via telephone does not prompt volunteers to leave their home, which reduces the likelihood of the often-stated increase of daily physical activity associated with other forms of volunteering (e.g., walking, climbing stairs). Instead of focusing on physical outcomes, the social outcomes, primarily loneliness, perceived social support, and network parameters are of interest and are complemented by several additional self-reports of mental well-being.

### 3.2 Conclusions

Not only does Hong Kong – like many industrialized societies – face major challenges for health care services and welfare due to its rapid demographic change [76], increased rates of self-reported loneliness across large parts of its population are observed as well [6]. If the delivery of our loneliness intervention via telephone is effective for intervention recipients and scalable – hence if its delivery reaches older adults experiencing loneliness, who live isolated from others, without internet, and with lower mobility – without the need for physical contact, this would be an important program for future times of crises. If our trial can show that volunteers delivering one of the telephone-based interventions to lonely intervention recipients benefit themselves from their voluntary intervention delivery, this could constitute a win-win situation that encourages older adults to engage in, and profit from, active ageing strategies.

If successful, this program has the potential for large-scale implementation. The program could be implemented by NGOs aiming to offer volunteering opportunities to older adults that face problems with mobility or those living in rural areas. The program could also be integrated into political efforts to prevent burdens on the health-care system, to ease societal tensions created by rapid demographic change, and to strengthen the third sector. The volunteering results of the HEAL-HOA trial look very promising after 6 months of follow-up [77].

## Ethics approval and consent to participate

Ethical approval for this RCT was obtained by the Human Research Ethics Committee (HREC) of the Hong Kong University of Education (Reference Number: 2019-2020-0442). The study was prospectively registered in the Clinical Trials Registry of the University of Hong Kong Clinical Trials Centre (number HKUCTR-2929; as well as retrospectively registered at the Chinese Clinical Trial Registry in the WHO Registry Network, number ChiCTR2300072909). The contents of the two registrations are identical.

## Availability of data and material

The datasets generated during the study will be available from Prof. Keelee Chou klchou@eduhk.hk upon reasonable request.

## Funding

The RCT is funded by the Research Grants Council of the Hong Kong Special Administrative Region Government (project number: C8105-20 GF), Research Grant Council of the Hong Kong Special Administrative Region Government.

## CRediT authorship contribution statement

**Lisa M. Warner:** Writing – original draft. **Da Jiang:** Methodology, Project administration, Resources, Supervision, Validation, Writing – review & editing, Conceptualization. **Dannii Yuen-lan Yeung:** Conceptualization, Methodology, Project administration, Resources, Supervision, Validation, Writing – review & editing. **Namkee G. Choi:** Conceptualization, Supervision, Validation, Writing – review & editing. **Rainbow Tin Hung Ho:** Conceptualization, Methodology, Project administration, Resources, Supervision, Validation, Writing – review & editing. **Jojo Yan Yan Kwok:** Conceptualization, Methodology, Project administration, Supervision, Validation, Writing – review & editing. **Youqiang Song:** Conceptualization, Supervision, Writing – review & editing. **Kee-Lee Chou:** Conceptualization, Funding acquisition, Methodology, Project administration, Resources, Supervision, Validation, Writing – review & editing.

## Declaration of competing interest

The authors declare that they have no known competing financial interests or personal relationships that could have appeared to influence the work reported in this paper.
